# Nonextensive Statistical Mechanics: Equivalence Between Dual Entropy and Dual Probabilities

**DOI:** 10.3390/e22060594

**Published:** 2020-05-26

**Authors:** George Livadiotis

**Affiliations:** Division of Space Science and Engineering, Southwest Research Institute, San Antonio, TX 78238, USA; glivadiotis@swri.edu; Tel.: +1-210-522-3415

**Keywords:** nonextensive statistical mechanics, kappa distributions, *q*-entropy, escort probability

## Abstract

The concept of duality of probability distributions constitutes a fundamental “brick” in the solid framework of nonextensive statistical mechanics—the generalization of Boltzmann–Gibbs statistical mechanics under the consideration of the *q*-entropy. The probability duality is solving old-standing issues of the theory, e.g., it ascertains the additivity for the internal energy given the additivity in the energy of microstates. However, it is a rather complex part of the theory, and certainly, it cannot be trivially explained along the Gibb’s path of entropy maximization. Recently, it was shown that an alternative picture exists, considering a dual entropy, instead of a dual probability. In particular, the framework of nonextensive statistical mechanics can be equivalently developed using *q*- and 1/*q*- entropies. The canonical probability distribution coincides again with the known *q*-exponential distribution, but without the necessity of the duality of ordinary-escort probabilities. Furthermore, it is shown that the dual entropies, *q*-entropy and 1/*q*-entropy, as well as, the 1-entropy, are involved in an identity, useful in theoretical development and applications.

## 1. Introduction

Non-extensive statistical mechanics generalizes the classical statistical framework of Boltzmann–Gibbs (BG). The generalization is based on two fundamental considerations, (i) the *q*-entropy, a monoparametrical generalization of BG’s entropy [1], and (ii) the escort probability distribution [2,3], a metastable distribution at which the ordinary distribution that maximizes the entropy is stabilized. The metastable distribution coincides with the empirical model of frequently observed distributions in nature and especially in plasmas, called either *q*-exponential or kappa distribution.

The *q-*exponential distribution, named after the “*q*-deformed exponential” function (e.g., [4,5]), is the non-extensive version of the canonical distribution, that is, a distribution governed by the flexible parameter *q* that maximizes the *q*-entropy under the constraints of the canonical ensemble. The *q*-exponential distributions are observed quite frequently in nature, and constitute a suitable generalization of the BG exponential distribution. Applications of the *q*-exponential distribution can be found in a wide variety of topics, among numerous others, are the following: sociology–sociometry: e.g., internet [6]; citation networks of scientific papers [7]; urban agglomeration [8]; linguistics [9]; economy [10]; biology: biochemistry [11,12]; ecology [13,14]; statistics: [15,16,17,18]; physics: e.g., nonlinear dynamics [19,20]; condensed-matter: [21]; earthquakes [22,23,24,25,26]; turbulence [27,28]; physical chemistry [29]; and space physics/astrophysics [30,31,32]; (a more extended bibliography of *q*-deformed exponential distributions can be found in [14,32,33,34,35,36,37]).

The connection of kappa distributions with non-extensive statistical mechanics, as well as the equivalence between the *q*-exponential and kappa distributions, has been examined by several authors (e.g., [37,38,39,40,41]). The empirical kappa distribution and the Tsallis-like Maxwellian distribution of velocities are accidentally of the same form, under a transformation between the *q*-index and the kappa parameter that labels and governs the kappa distributions. (For details on this topic, see the review [41], the special issue introduction [42], and the book of kappa distributions: [32]). Understanding the statistical origin of these distributions was a cornerstone of theoretical developments and a plethora of applications in space plasma physics and complexity science.

The kappa distributions were found to describe the particle velocities in various space plasma analyses: (i) the inner heliosphere, including solar wind (e.g., [43,44,45,46,47,48,49,50,51,52,53,54,55,56,57,58]), solar spectra (e.g., [59,60,61]), solar corona (e.g., [62,63,64,65]), solar energetic particles (e.g., [66,67,68]), corotating interaction regions (e.g., [69,70]), and solar flares related (e.g., [71,72,73,74,75]), shocks (e.g., [70,76]); (ii) the planetary magnetospheres, including magnetosheath (e.g., [77,78,79,80]), near magnetopause (e.g., [81]), magnetotail (e.g., [82]), ring current (e.g., [83]), plasma sheet (e.g., [84,85]), magnetospheric substorms (e.g., [86,87]), Aurora (e.g., [86,88]), magnetospheres of giant planets, such as Jovian (e.g., [89,90,91,92]), Saturnian (e.g., [93,94,95,96,97]), Uranian (e.g., [98]), Neptunian (e.g., [99]), magnetospheres of planetary moons, such as Io (e.g., [100]) and Enceladus (e.g., [101]), or cometary magnetospheres (e.g., [102]); (iii) the outer heliosphere and the inner heliosheath (e.g., [41,52,103,104,105,106,107,108,109,110,111,112,113,114,115,116,117,118,119,120]); (iv) beyond the heliosphere, including stellar spectra (e.g., [121]), HII regions (e.g., [122]), planetary nebula (e.g., [122,123,124]], supernova magnetospheres (e.g., [125]), dark matter (e.g., [126]), and in cosmological scales (e.g., [127]).

It is now well understood that the previously mentioned examples of observed distributions can be described by the *q*-exponential or kappa distributions, that is, the type of distribution maximizing the *q*-entropy in the canonical ensemble.

It was recently shown that the statistical framework of non-extensive statistical mechanics could be deduced as it is, without the consideration of the dual formalism of ordinary/escort distributions. This concept can significantly simplify the usage of the theory, and make it accessible by the new generation of researchers that are straggling to understand and apply it in exotic particle systems out of thermal equilibrium, such as the space plasmas. However, the cost of this simplification is the necessity of having two types of entropic functions. This dual formulation preserves the basic fundamental thermodynamic formulae, which is necessary for the consistent connection of the statistical mechanics with the thermodynamics. In Section 2, the paper presents the standard nonextensive statistical mechanics, which is based on escort duality formalism (that involves the maximization of *q*-Entropy using the ordinary/escort duality formalism). In Section 3, we show how the framework of nonextensive statistical mechanics can be developed, considering entropy metastable duality. The paper deals with the duality between the *q*- and 1/*q*- entropies; the maximization of the latter under the canonical ensemble; the derivation of an identity formula involving the dual entropies, *q*-entropy and 1/*q*-entropy, as well as, the 1-entropy, which can be useful in theoretical development and applications. As an example, in Section 4, we focus on the continuous description of energy distribution. Finally, Section 5 summarizes the conclusions.

## 2. Nonextensive Statistical Mechanics with Probability Metastable Duality

### 2.1. q-Entropy and Ordinary/Escort Duality Formalism

Consider the discrete energy spectrum ${\left\{\varepsilon_{k}\right\}}_{k=1}^{W}$ associated with a discrete probability distribution ${\left\{p_{k}\right\}}_{k=1}^{W}$. Non-extensive statistical mechanics is based on the *q*-entropy and the dual formalism of ordinary/escort probabilities. The non-extensive entropy is given by [1]:

(1) $$S_{q}=\frac{1}{q-1}\cdot \left(1-\sum_{k=1}^{W}p_{k}{}^{q}\right),$$

leading to the BG formulation for *q* → 1, $S_{1}=-\sum_{k}^{W}p_{k}\ln(p_{k})$ (note: the entropic formulations are given in units of the Boltzmann’s constant *k*_B_).

On the other hand, the escort probability distribution ${\left\{P_{k}\right\}}_{k=1}^{W}$ is constructed from the ordinary probability distribution, ${\left\{p_{k}\right\}}_{k=1}^{W}$, as follows [2]:

(2) $$P_{k}=\frac{p_{k}{}^{q}}{\sum_{k=1}^{W}p_{k}{}^{q}}\Leftrightarrow p_{k}=\frac{P_{k}{}^{1/q}}{\sum_{k=1}^{W}P_{k}{}^{1/q}},\;\mathrm{for}\;\forall k=1,\ldots ,W.$$

### 2.2. Maximization of q-Entropy

The maximization of entropy is derived from $(\partial /\partial p_{j})S_{q}\left({\left\{p_{k}\right\}}_{k=1}^{W}\right)=0$, ∀ j=1,…,W. However, the probabilities ${\left\{p_{k}\right\}}_{k=1}^{W}$ do not constitute independent variables, because of the constraints of (i) probability normalization, and (ii) fixed internal energy; the two constraints can be expressed either in terms of the ordinary or the escort probabilities, i.e.,

(3) $$(i)\;\sum_{k=1}^{W}p_{k}=1,\;(\mathrm{ii})\;\sum_{k=1}^{W}p_{k}{}^{q}\;\varepsilon_{k}/\sum_{k=1}^{W}p_{k}{}^{q}=U,\;\mathrm{or},\;$$

(4) $$(i)\;\sum_{k=1}^{W}P_{k}=1,\;(\mathrm{ii})\;\sum_{k=1}^{W}P_{k}\;\varepsilon_{k}=U.\;$$

The Lagrange method (as used by Gibbs [128]) involves maximizing an alternative functional *G*, instead of the entropy *d* directly, that is, finding $(\partial /\partial p_{j})G\left({\left\{p_{k}\right\}}_{k=1}^{W}\right)=0$, ∀ j=1,…,W, where *G* is written in terms of the Lagrange multipliers $\lambda_{1}$ and $\lambda_{2}$ by

(5) $$G\left({\left\{p_{k}\right\}}_{k=1}^{W}\right)=S_{q}\left({\left\{p_{k}\right\}}_{k=1}^{W}\right)+\lambda_{1}\cdot 1+\lambda_{2}\cdot U,\;\mathrm{or}\;$$

(6) $$G\left({\left\{p_{k}\right\}}_{k=1}^{W}\right)=\frac{1}{q-1}\cdot \sum_{k=1}^{W}\left(p_{k}-p_{k}{}^{q}\right)+\lambda_{1}\sum_{k=1}^{W}p_{k}+\lambda_{2}\sum_{k=1}^{W}p_{k}{}^{q}\;\varepsilon_{k}/\sum_{k=1}^{W}p_{k}{}^{q}.$$

The maximization of this functional leads to the ordinary probability distribution

(7) $$p_{j}=\frac{1}{Z_{q}}\cdot {\left[1-\left(1-q\right)\beta (\varepsilon_{j}-U)\right]}_{}^{\;\frac{1}{1-q}},\;Z_{q}\equiv \sum_{j=1}^{W}{\left[1-\left(1-q\right)\beta (\varepsilon_{j}-U)\right]}_{}^{\;\frac{1}{1-q}}.$$

The multiplier $\lambda_{1}$ is connected to the partition function $Z_{q}\equiv {\left(\lambda_{1}\cdot (q-1)/q\right]}^{1/(q-1)}$. The other multiplier, $\lambda_{2}$, is connected to temperature $(k_{B}T)\propto -\;\lambda_{2}{}^{-1}$. In particular, the negative and inverse value of the second Lagrange multiplier defines the so-called Lagrangian temperature *T*_L_ [114], i.e.,

(8) $$T\equiv T_{L}\cdot \phi_{q},\;\mathrm{with}\;\phi_{q}\equiv \sum_{k=1}^{W}p_{k}{}^{q}=Z_{q}{}^{1-q}.$$

Substituting Equation (8) into Equation (2) leads to the escort distribution:

(9) $$P_{j}(\varepsilon_{j})=\frac{1}{Z_{q}}\cdot \;{\left[1+(q-1)\cdot \beta (\varepsilon_{j}-U)\right]}_{}^{\;-\;\frac{1}{q-1}-1}$$

The escort probability distribution describes a metastable distribution at which the ordinary distribution that maximizes the entropy is duplexed. The metastable distribution coincides with the kappa distribution, under the transformation of the kappa and *q* indices [37]:

(10) $$\kappa =\frac{1}{q-1}\Leftrightarrow q=1+\frac{1}{\kappa },$$

leading to the kappa distribution, in the discrete description,

(11) $$P_{j}(\varepsilon_{j})\propto \;{\left[1+\frac{1}{\kappa }\cdot \beta (\varepsilon_{j}-U)\right]}_{}^{\;-\kappa -1}.$$

Or, in the continuous description,

(12) $$P(\varepsilon )\propto \;{\left[1+\frac{1}{\kappa }\cdot \beta (\varepsilon -U)\right]}_{}^{\;-\kappa -1}.$$

## 3. Nonextensive Statistical Mechanics with Entropy Metastable Duality

### 3.1. Entropy Duality Formalism

Alternatively, the exactly identical framework of non-extensive statistical mechanics can be developed by considering a dual entropy, instead of a dual probability distribution (as it was considered in Section 2).

The duality is given by the standard *q*-entropy and the one with inverse *q*-index, i.e., (1/*q*)-entropy. In order to show this, we accept that there is only one type of distribution; this must coincide with the metastable distribution of the case where the duality in probabilities was considered (Section 2); that is, the escort probability distribution. Then, the 1/*q*-entropy is expressed by [129]:

(13) $${\tilde{S}}_{1/q}=\frac{1}{1/q-1}\cdot \left(1-\sum_{k=1}^{W}P_{k}{}^{1/q}\right),$$

(notice the usage of escort instead of the ordinary probability distribution). Using the entropy *S_q_* in Equation (1) and the following identity that can be easily derived from Equation (2):

(14) $$\sum_{k=1}^{W}P_{k}{}^{1/q}={\left(\sum_{k=1}^{W}p_{k}{}^{q}\right)}^{-1/q},$$

we have

(15) $${\tilde{S}}_{1/q}=\frac{1}{1/q-1}\cdot \left[1-{\left(\sum_{k=1}^{W}p_{k}{}^{q}\right)}^{-1/q}\right]\Rightarrow 1-(1/q-1){\tilde{S}}_{1/q}={\left[1-(q-1)S_{q}\right]}^{\;-\;\frac{1}{q}},$$

leading to

(16) $${\left[1-(1/q-1){\tilde{S}}_{1/q}\right]}^{\;-\;\frac{1}{1\;/q-1}}={\left[1-(q-1)S_{q}\right]}^{\;-\;\frac{1}{q-1}}=Z_{q}.$$

We observe that there are always two different indices, *q* and 1/*q*, (except for the case of *q* = 1 when both indices coincide), for which the partition function $Z_{Q}={\left[1-(Q-1)S_{Q}\right]}^{\;-\;\frac{1}{Q-1}}$ remains invariant for the two indices, $Q_{1}=q$ and $Q_{2}=1/q$; however, one has to recall that ${\tilde{S}}_{Q}\neq S_{Q}$ for any Q≠1.

### 3.2. q-Deformed Exponential/Logarithm Functions

The *Q*-deformed exponential function [4,5] and the *Q*-logarithm function, are defined by

(17) $$\exp_{Q}(x)={\left[1+(1-Q)\cdot x\right]}_{+}^{-\;\frac{1}{Q-1}},\;\ln_{Q}(x)=\frac{1-x^{1-Q}}{Q-1},\;$$

where the subscript “+” denotes the cut-off condition, where $\exp_{Q}(x)$ becomes zero if its base is non-positive. These are inverse functions for any *Q* (in similar to the case of *Q* = 1):

(18) $$\exp_{Q}[\ln_{Q}(x)]=\ln_{Q}[\exp_{Q}(x)]=1.\;$$

Hence, the dual ordinary/escort distributions are written as:

(19) $$p_{j}(\varepsilon_{j})\propto \exp_{q}^{}\;{\left[-\beta (\varepsilon_{j}-U)\right]}^{}\Leftrightarrow P_{j}(\varepsilon_{j})\propto \exp_{q}^{q}\;\left[-\beta (\varepsilon_{j}-U)\right],\;$$

while the dual *q*- / (1/*q*)- entropies in Equation (16) are written as:

(20) $$\exp_{1/q}({\tilde{S}}_{1/q})=\exp_{q}(S_{q}).\;$$

### 3.3. Maximization of 1/q-Entropy

The maximization of 1/*q*-entropy leads directly to the escort probability distribution. Indeed, by maximizing the functional *G*, as in Equation (6),

(21) $$G\left({\left\{P_{k}\right\}}_{k=1}^{W}\right)=\frac{1}{(1/q)-1}\cdot \sum_{k=1}^{W}\left(P_{k}-P_{k}{}^{1/q}\right)+\lambda_{1}\sum_{k=1}^{W}P_{k}+\lambda_{2}\sum_{k=1}^{W}P_{k}\varepsilon_{k},\;$$

we find [129,130,131]:

(22) $$P_{j}(\varepsilon_{j})=\frac{1}{Z_{q}}\cdot {\left[\;1+(q-1)\cdot \beta (\varepsilon_{j}-U)\right]}_{}^{\;-\;{\frac{q}{q-1}}_{}}=\frac{1}{Z_{q}}\cdot \exp_{q}^{q}\left[-\beta (\varepsilon_{j}-U)\right],\;$$

or, in terms of the kappa index (via Equation (10)):

(23) $$P_{j}(\varepsilon_{j})=\frac{1}{Z_{q}}\cdot {\left[\;1+\frac{1}{\kappa }\cdot \beta (\varepsilon_{j}-U)\right]}_{}^{\;-\kappa -1}.\;$$

It has to be stressed out that (i) the correct canonical distribution is derived without the duality of ordinary/escort distributions, since only one distribution is considered; however, (ii) the canonical distribution in Equation (23) is not derived by maximizing the system’s *q*-entropy Sq; instead, it is deduced by maximizing the (1/*q*)-entropy, $\tilde{S}1/q$, the dual of the system’s *q*-entropy.

### 3.4. q-Independent Information Measure

In [131], we examine the thermodynamic origin of *q*-entropy and its associated *q*-exponential or kappa distributions. As it was shown, the classical concept of thermal equilibrium and the thermodynamic definition of temperature, given by

(24) $$\frac{1}{T}=\frac{\partial S_{1}}{\partial U},\;$$

can be naturally generalized to

(25) $$\frac{1}{T}=\frac{\partial }{\partial U}\ln\left[\exp_{q}(S_{q})\right].$$

The classical BG entropy is noted as *S*_1_, that is, the *q*-entropy *S_q_* for *q* = 1. When *q* = 1, the expression ln[expq (Sq)] at the right-hand-side of Equation (25) becomes simply *S*_1_. On the other hand, the internal energy does not depend on the *q*- or kappa indices; for example, in the continuous case we have $U=\frac{1}{2}f\;k_{B}T$. This is because the kappa index is irrelevant to the energy transition among particles, but is count only for the correlation among particle energies. In addition, the temperature and kappa index are found to be two independent thermodynamic variables. Therefore, if the temperature and internal energy are quantities independent of the *q*-index, then, the quantity ln[expq (Sq)] should be a sum of a *q*-dependent function, lng(q), and a U-dependent function, f(U), i.e.,

(26) $$\ln[\exp_{q}(S_{q})]=\ln g(q)+f(U)=\ln g(q)+S_{1},\;$$

where we set the value of lng(1) to be absorbed by f(U), and thus we may redefine function *g* to be lng(1)=0, or $S_{1}=f(U)$. Hence,

(27) $$\exp_{q}(S_{q})=g(q)\cdot \exp(S_{1}).$$

Finally, Equation (27) completes the duality in Equation (20), ending up with

(28) $$\exp_{1/q}({\tilde{S}}_{1/q})=g(q)\cdot \exp(S_{1})=\exp_{q}(S_{q}).\;$$

## 4. Application in the Continuous Description

Extensivity requires that the entropy of the whole system is proportional to the size of the system or the number of independent particles of the system. Additivity means that the entropy of the whole system sums up the entropies of all the statistically independent subsystems. Apparently, additivity leads to extensivity, but non-additivity does not mean non-extensivity. Macroscopically all physically meaningful entropies end up to be extensive (as the number of particles tends to infinity). This can be understood as follows. Depending on the range of interactions whether it is small or long, there is always a scale—let this be λ_C_—in which particles are characterized by local correlations. Particles within this scale are correlated to each other, but the particles from different particles are non-correlated, i.e., independent. Let *N*_C_ be the number within the scale λ_C_. If *N* is the total number of particles, then, there is about *M*~*N*/*N*_C_ uncorrelated groups of correlated particles of length λ_C_. Since there is no correlation among all *M* groups, the total entropy of the system is similar to BG statistical mechanics, that is, *S* = *M∙S*_C_, where *S*_C_(*N*_C_) is the entropy characterizing the scale in which particles are correlated; this is rewritten as *S* = *N∙S_q_*, where *S_q_*(*N*_C_) is the per particle entropy that characterizes the scale λ_C_, while it depends in a nonlinear way on the number of *N*_C_ particles; therefore, the entropy of the whole system *S* is macroscopically proportional to the number of its particles *N*, independently of the number *N*_C_. For instance, Boltzmann–Gibbs (BG) statistics considers no correlations among particles [128], that is, *N*_C_ = 1. On the other hand, nonextensive statistics for plasmas considers local correlations among particles, with a typically large number *N*_C_, given by the number within a Debye sphere, *N*_D_. (For more details, e.g., see: [32].)

As an example, we show the continuous description of kappa distributions. The kappa index depends on the total number of correlated degrees of freedom f.

The physical meaning of the kappa index is the reciprocal correlation coefficient of the energies of any two correlated kinetic degrees of freedom. In particular, the correlation coefficient is given by *ρ* = (3/2)/*κ* [112]. The kappa index *κ* is dependent on the correlated degrees of freedom *f*, and can be related to an invariant kappa index $\kappa_{0_{}}$ by $\kappa (f)=\kappa_{0_{}}+(1/2)f$. For a number of *N*_C_ correlated particles with *d* degrees of freedom per particle, we have $f=d\cdot N_{C}$, and the dependent kappa index is $\kappa (N_{C})=\kappa_{0_{}}+(d/2)N_{C}$. Note that $\kappa_{0_{}}$ is the actual kappa index that characterizes a stationary state, and it is invariant from the number of particles and degrees of freedom of the system [112].

The corresponding partition function *Z_q_* was found to be [114]:

(29) $$Z_{q}={\left(\frac{\sigma^{2}}{\pi \;e}\right)}^{-\frac{1}{2}d\cdot N_{C}}\cdot \frac{\Gamma (\kappa_{0})\cdot {\left(\kappa_{0}/e\right)}^{-\kappa_{0}}}{\Gamma (\kappa_{0}+\frac{d}{2}\cdot N_{C})\cdot {\left[(\kappa_{0}+\frac{d}{2}\cdot N_{C})/e\right]}^{\;-(\kappa_{0}+\frac{1}{2}d\cdot N_{C})}},$$

where *N*_C_ is the number of correlation particles included in a correlation length $\ell_{C}$; the involved dimensionless scale parameter σ is expressed in terms of the thermal speed $\theta =\sqrt{2k_{B}T/\;m\;}$ for particles of mass *m*, and the *d*-dim spherical volume of radius equal to the correlation length, i.e., $V_{C}=v_{d}\cdot \ell_{C}^{\;d}$ with $v_{d}^{}=\pi^{d/2}/[(\frac{d}{2})!]$, that is,

(30) $$\sigma =\frac{h}{m\theta \;{\left(V_{C}\right)}^{1/d}},$$

or, considering an ion-electron plasma, with masses *m_i_* and *m_e_*, and temperatures *T_i_* and *T_e_*,

(31) $$\sigma^{2}=2\;\pi {\left[(\frac{d}{2})!\right]}^{2/d}\frac{\hbar^{2}}{k_{B}\sqrt{T_{i}T_{e}m_{i}m_{e}\;}\;\ell_{C}^{\;2}}.$$

Therefore, the partition function becomes:

(32) $$Z_{q}={\left\{\frac{\frac{2}{e}{\left[(\frac{d}{2})!\right]}^{2/d}\;\hbar^{2}}{k_{B}\sqrt{T_{i}T_{e}m_{i}m_{e}\;}\;\ell_{C}^{\;2}}\right\}}^{-\frac{1}{2}d\cdot N_{C}}\cdot \frac{\Gamma (\kappa_{0})\cdot {\left(\kappa_{0}/e\right)}^{-\kappa_{0}}}{\Gamma (\kappa_{0}+\frac{d}{2}\cdot N_{C})\cdot {\left[(\kappa_{0}+\frac{d}{2}\cdot N_{C})/e\right]}^{\;-(\kappa_{0}+\frac{1}{2}d\cdot N_{C})}},$$

or

(33) $$\begin{array}{c} \ln Z_{q}=\frac{1}{2}d\cdot N_{C}\ln\left\{\frac{k_{B}\sqrt{T_{i}T_{e}m_{i}m_{e}\;}\;\ell_{C}^{\;2}}{\frac{2}{e}{\left[(\frac{d}{2})!\right]}^{2/d}\;\hbar^{2}}\right\}\\ +\ln\left\{\frac{\Gamma (\kappa_{0})\cdot {\left(\kappa_{0}/e\right)}^{-\kappa_{0}}}{\Gamma (\kappa_{0}+\frac{d}{2}\cdot N_{C})\cdot {\left[(\kappa_{0}+\frac{d}{2}\cdot N_{C})/e\right]}^{\;-(\kappa_{0}+\frac{1}{2}d\cdot N_{C})}}\right\}. \end{array}$$

We observe that Equation (33) has exactly the form of Equation (26)! Namely,

(34) $$\ln Z_{q}=\ln g(q)+S_{1},\;\mathrm{or},\;Z_{q}=e^{S_{1}}\cdot g(q),\;$$

with

(35) $$g(q=1+1/\kappa )=\frac{\Gamma (\kappa_{0})\cdot {\left(\kappa_{0}/e\right)}^{-\kappa_{0}}}{\Gamma (\kappa_{0}+\frac{d}{2}\cdot N_{C})\cdot {\left[(\kappa_{0}+\frac{d}{2}\cdot N_{C})/e\right]}^{\;-(\kappa_{0}+\frac{1}{2}d\cdot N_{C})}},$$

and lng(1)=0, while *S*_1_ equals the Sackur–Tetrode entropic formula [72,114],

(36) $$S_{1}=\frac{1}{2}d\cdot N_{C}\ln\left\{\frac{k_{B}\sqrt{T_{i}T_{e}m_{i}m_{e}\;}\;\ell_{C}^{\;2}}{\frac{2}{e}{\left[(\frac{d}{2})!\right]}^{2/d}\;\hbar^{2}}\right\},\;\mathrm{or},\;e^{\frac{1}{\frac{1}{2}d\cdot N_{C}}\cdot S_{1}}=\frac{k_{B}\sqrt{T_{i}T_{e}m_{i}m_{e}\;}\;\ell_{C}^{\;2}}{\frac{2}{e}{\left[(\frac{d}{2})!\right]}^{2/d}\;\hbar^{2}}.$$

Solving in terms of entropies, Equation (28) gives

(37) $${\tilde{S}}_{1/q}=\ln_{1/q}(Z_{q})=\frac{1-Z_{q}{}^{\;1-1\;/q}}{1/q-1},\;S_{q}=\ln_{q}(Z_{q})=\frac{1-Z_{q}{}^{\;1-q}}{q-1},$$

or, in terms of the kappa index,

(38) $${\tilde{S}}_{1/q}=(\kappa +1)\cdot \left(Z_{q}{}^{\;\frac{1}{\kappa +1}}-1\right)=(1+\kappa_{0}+\frac{d}{2}\cdot N_{C})\cdot \left(Z_{q}{}^{\;\frac{1}{1+\kappa_{0}+\frac{d}{2}\cdot N_{C}}}-1\right),$$

(39) $$S_{q}=\kappa \cdot \left(1-Z_{q}{}^{\;-\frac{1}{\kappa }}\right)=(\kappa_{0}+\frac{d}{2}\cdot N_{C})\cdot \left(1-Z_{q}{}^{\;-\frac{1}{\kappa_{0}+\frac{d}{2}\cdot N_{C}}}\right).$$

Substituting *Z_q_* from Equations (34,35) into Equations (38,39), we end up with

(40) $$\begin{array}{c} {\tilde{S}}_{1/q}=(1+\kappa_{0}+\frac{d}{2}\cdot N_{C})\\ \times \;\left\{{\left[e^{S_{1}}\cdot \frac{\Gamma (\kappa_{0})\cdot {\left(\kappa_{0}/e\right)}^{-\kappa_{0}}}{\Gamma (\kappa_{0}+\frac{d}{2}\cdot N_{C})\cdot {\left[(\kappa_{0}+\frac{d}{2}\cdot N_{C})/e\right]}^{\;-(\kappa_{0}+\frac{1}{2}d\cdot N_{C})}}\right]}^{\;\frac{1}{1+\kappa_{0}+\frac{d}{2}\cdot N_{C}}}-1\right\}\;, \end{array}$$

(41) $$\begin{array}{c} S_{q}=(\kappa_{0}+\frac{d}{2}\cdot N_{C})\\ \times \;\left\{1-{\left[e^{S_{1}}\cdot \frac{\Gamma (\kappa_{0})\cdot {\left(\kappa_{0}/e\right)}^{-\kappa_{0}}}{\Gamma (\kappa_{0}+\frac{d}{2}\cdot N_{C})\cdot {\left[(\kappa_{0}+\frac{d}{2}\cdot N_{C})/e\right]}^{\;-(\kappa_{0}+\frac{1}{2}d\cdot N_{C})}}\right]}^{\;-\;\frac{1}{\kappa_{0}+\frac{d}{2}\cdot N_{C}}}\right\}\;. \end{array}$$

As noted by [129], Sq entropy has a fine property that lacks in $\tilde{S}1/q$: at conditions near the classical thermal equilibrium of large values of *κ*_0_ and *S*_1_, the slope of entropy must be positive, so that, the closer to the classical equilibrium (*κ*_0_→∞), the higher the entropy. We have:

(42) $${\frac{\partial S_{q}}{\partial \kappa_{0}}|}_{\;S_{1}>>1}>0,\;{\frac{\partial {\tilde{S}}_{1/q}}{\partial \kappa_{0}}|}_{\;S_{1}>>1}<0.$$

**Figure 1:**
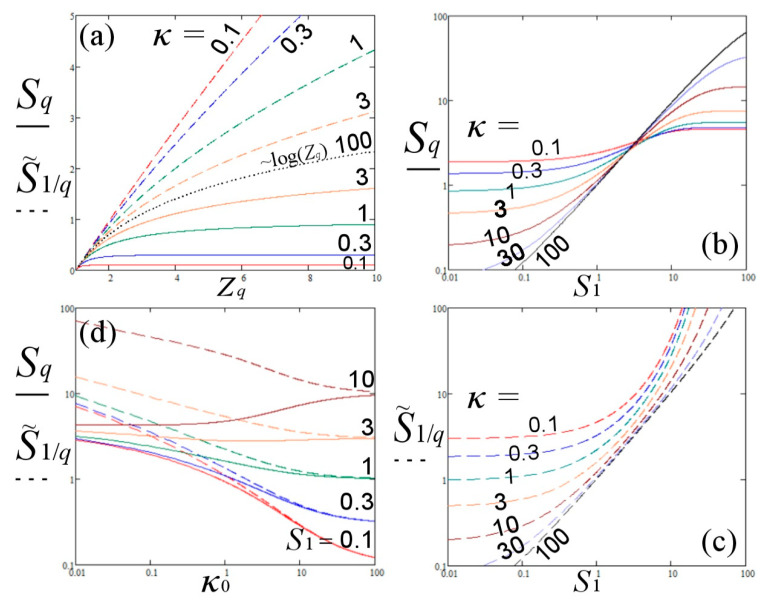
(**a**) Entropies Sq and $\tilde{S}1/q$ plotted as a function of $Z_{q}$, as shown in Equation (37), for various kappa indices; the case of *κ*→∞ corresponding to log(*Z_q_*) is shown. Entropies (**b**) Sq, and (**c**) $\tilde{S}1/q$, plotted as a function of *S*_1_, as shown in Equations (38,39), for various kappa indices. (**d**) Entropies Sq and $\tilde{S}1/q$ plotted as a function of the invariant kappa index *κ*_0_, as shown in Equations (40,41), for various values of the BG entropy, *S*_1_; we observe that (i) the two entropies tend to *S*_1_ as *κ*_0_→∞; for large values of *S*_1_, entropy Sq has a positive slope, while entropy $\tilde{S}1/q$ has negative slope (a nonrealistic property).

In Figure 1, we plot the entropies Sq and $\tilde{S}1/q$ as a function of (a) $Z_{q}$, (b,c) *S*_1_, and (d) *κ*_0_.

These results verify that the entropy of the particle system is given by Sq, and not by $\tilde{S}1/q$, though, it is the entropic function of $\tilde{S}1/q$, which is maximized to lead to the canonical distribution *P*(*ε*). Therefore, the standard description of nonextensive statistical mechanics considers the entropy Sq, which is maximized to provide *p*(*ε*), and indirectly, the dual escort distribution *P*(*ε*), i.e., the actual distribution *P*(*ε*) is dual to the auxiliary distribution *p*(*ε*) that comes from the maximization of entropy. In the present picture of nonextensive statistical mechanics, the entropy has the duality property, and not the distribution, i.e., the actual Sq is dual to the auxiliary entropy $\tilde{S}1/q$ that needs to be maximized.

## 5. Conclusions

The paper is a theoretical analysis on the dualities that characterize nonextensive statistical mechanics. The concept of duality of probability distributions is of fundamental importance within the framework of nonextensive statistical mechanics—the generalization of Boltzmann–Gibbs statistical mechanics under the consideration of the *q*-entropy. While the probability duality is solving old-standing issues of the theory, e.g., it ascertains the additivity for the internal energy given an additivity in the energy of microstates, is a rather complex part of the theory, and certainly, it cannot be trivially explained along the Gibb’s path of entropy maximization.

Recently, it was shown that an alternative picture exists, considering a dual entropy, instead of a dual probability. In particular, the framework of nonextensive statistical mechanics can be equivalently developed using *q*- and 1/*q*- entropies (noted by Sq and $\tilde{S}1/q$). The canonical probability distribution coincides again with the known *q*-exponential distribution, but without the necessity of the duality of ordinary-escort probabilities. The paper deals with this duality between the *q*- and 1/*q*- entropies; the maximization of the 1/*q*- entropy under the canonical ensemble; the derivation of an identity formula involving the dual entropies, *q*-entropy and 1/*q*-entropy, as well as, the 1-entropy, which can be useful in theoretical development and applications.

It was shown that the entropy of the particle system is given by Sq and not by $\tilde{S}1/q$, though, it is the entropic function of $\tilde{S}1/q$ that should be maximized to lead directly to the canonical distribution *P*(*ε*). Therefore, the standard description of nonextensive statistical mechanics considers the entropy Sq, which is maximized to provide *p*(*ε*), and indirectly, the dual escort distribution *P*(*ε*), i.e., the actual distribution *P*(*ε*) is dual to the auxiliary distribution *p*(*ε*) that comes from the maximization of entropy. In the present picture of nonextensive statistical mechanics, the entropy has the duality property, and not the distribution, i.e., the actual Sq is dual to the auxiliary entropy $\tilde{S}1/q$ that is maximized to provide the canonical ensemble.

The paper focused on the continuous description of energy distribution. We show that the actual entropy of the system, Sq, can be expressed as a function of the kappa index, the number of particles, and the BG entropy, *S*_1_. At conditions near the classical thermal equilibrium, i.e., at large values of *κ*_0_ and *S*_1_, the slope of entropy is positive, verifying that the closer to the classical equilibrium (*κ*_0_ → ∞), the higher the value of the entropy, Sq.
